# Adenocarcinoma of the third and fourth portion of the duodenum: a case report and review of the literature

**DOI:** 10.1186/1757-1626-1-98

**Published:** 2008-08-18

**Authors:** Haridimos Markogiannakis, Dimitrios Theodorou, Konstantinos G Toutouzas, Georgia Gloustianou, Stilianos Katsaragakis, Ioannis Bramis

**Affiliations:** 1 1st Department of Propaedeutic Surgery, Hippokrateion Hospital, Athens Medical, School, University of Athens, Q. Sofias 114 av., 11527, Athens, Greece; 2 Department of Histopathology, Athens, Greece

## Abstract

A 65-year-old woman presented with abdominal pain, weight loss, fatigue, and microcytic anemia. Esophagogastroduodenoscopy, until the second part of duodenum, was normal. Ultrasound and computed tomography demonstrated a solid mass in the distal duodenum. A repeat endoscopy confirmed an ulcerative, intraluminar mass in the third and fourth part of the duodenum. Segmental resection of the third and fourth portion of the duodenum was performed. Histology revealed an adenocarcinoma. On the 4^th ^postoperative day, the patient developed severe acute pancreatitis leading to multiple organ failure and died on the 30^th ^postoperative day.

## Introduction

Although the small intestine constitutes over 75% of the length and 90% of the mucosal surface of the gastrointestinal tract, small intestine cancer is rare and accounts for only 1% of gastrointestinal malignancies [1,2]. Adenocarcinoma together with carcinoid tumours are the most common histological types of primary malignant tumours of the small bowel but other, including lymphoma and leiomyosarcoma, may less frequently be encountered [1,2]. Adenocarcinomas are predominantly located in the duodenum [1,2].

Duodenal adenocarcinomas represent approximately 0.3% of all malignant gastrointestinal tumours and the vast majority of them is found in the second portion of the duodenum [1,2]. Adenocarcinomas of the third and/or fourth portion of the duodenum, however, are very rare [1,2]. A case of adenocarcinoma of the third and fourth part of the duodenum is presented along with a literature review.

## Case presentation

**Figure 1:**
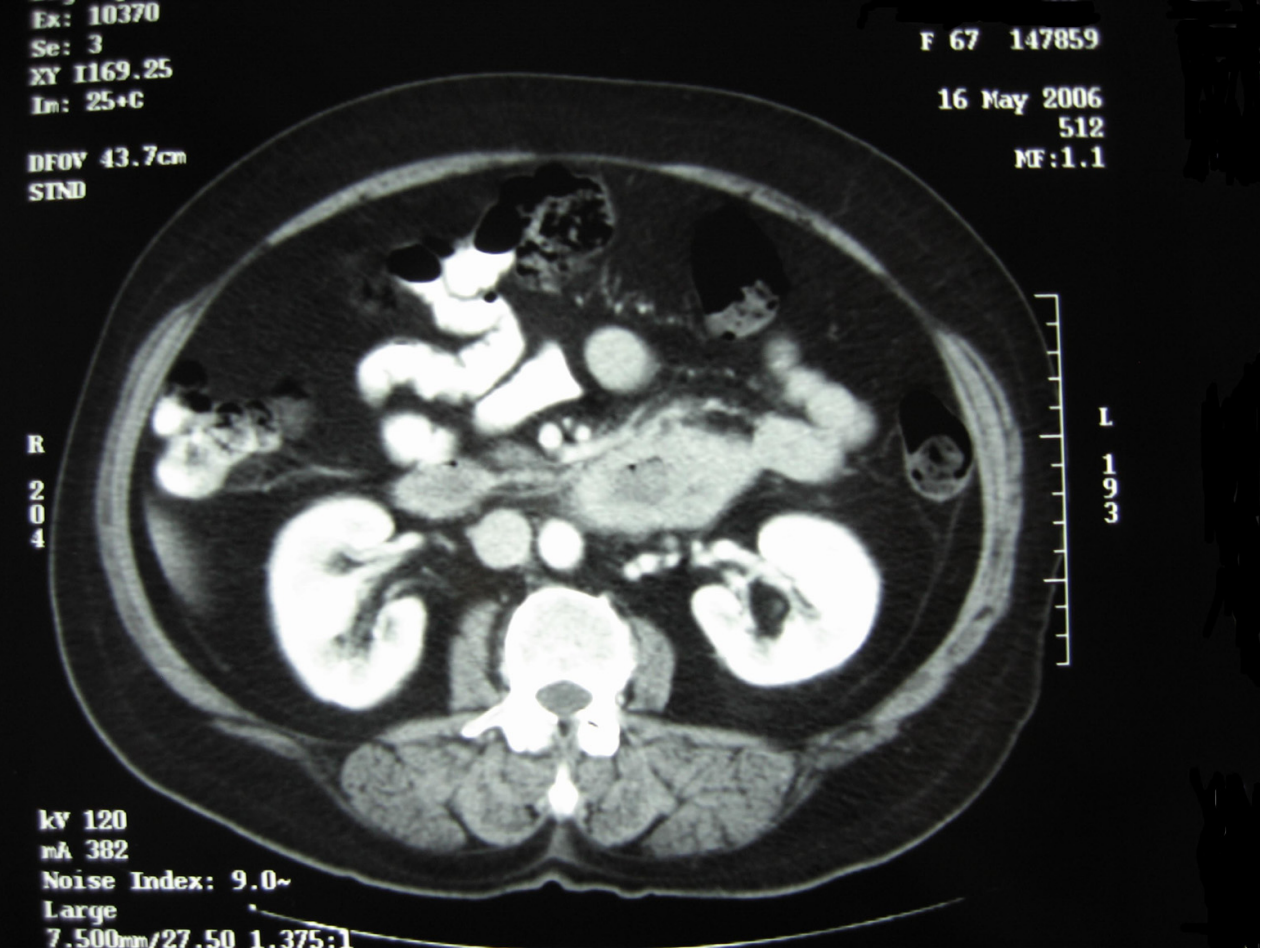
**Abdominal contrast-enhanced CT scan revealing a mass in the third and fourth part of the duodenum**.

A 65-year-old Greek woman (weight: 80 kgr, height: 160 cm) with a free past and family history, presented with a two month history of intermittent abdominal pain, weight loss, and fatigue. The patient had two normal labours, while she did not smoke, consume alcohol or take any medication. Clinical examination was normal. Blood tests, including tumour markers such as carcinoembryonic antigen (CEA), a-fetoprotein (AFP), carbohydrate antigen 19-9 (CA 19-9), and carbohydrate antigen 125 (CA 125), were within normal limits apart from microcytic anemia (hemoglobin: 8.0 gr/dl). Esophagogastroduodenoscopy, until the second portion of the duodenum, was normal. Abdominal ultrasound (US) finding of a hypoechoic mass with irregular margins in the distal duodenum led to a contrast-enhanced abdominal computed tomography (CT) scan that revealed a solid mass (6 × 5 cm) in the third and fourth part of the duodenum (Figure 1). No proximity to the stomach, pancreas, second portion of the duodenum, duodenojejunal flexure, proximal jejunum or colon was demonstrated. Portal vein, celiac axis, superior mesenteric artery and vein, pancreatic and bile duct were also free of tumour. No lymph node or distant metastasis was identified. Due to US and CT findings, a second endoscopy was performed which confirmed an ulcerative, intraluminar mass in the third and fourth part of the duodenum. Histology demonstrated an adenocarcinoma.

**Figure 2:**
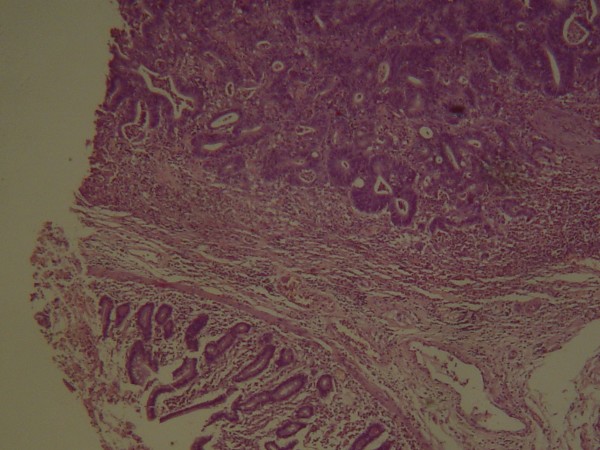
**Histopathology verified infiltration of the duodenal wall by an adenocarcinoma**. (HEX25).

Intraoperatively, a solid mass in the third and fourth part of the duodenum was identified. Local resectability of the tumour was meticulously investigated. Kocher's manoeuvre, mobilisation of the large intestine from the cecum to the midpoint of the transverse colon, mobilisation of the small bowel mesentery, division of the ligament of Treitz, and mobilisation of the third and fourth part of the duodenum along with the duodenojejunal flexure and proximal jejunum was performed. The stomach, pancreas, second portion of the duodenum, duodenojejunal flexure, proximal jejunum, and colon were free of tumour. Portal vein, celiac axis, superior mesenteric artery and vein, pancreatic and bile duct were also free of tumour. No lymph node or distant metastasis was identified. The neoplasm was, thus, considered resectable and a segmental resection of the third and fourth portion of the duodenum along with regional mesentery was performed. Intestinal continuity was then restored by an end-to-end hand sewn duodenojejunal anastomosis. Histopathologic evaluation of the resected specimen verified a moderately differentiated adenocarcinoma, measuring approximately 9 × 6 cm, infiltrating the duodenal wall, without any lymph node involvement (T_3_N_0_M_0_) (Figure 2). The specimen's margins were free of tumour.

On the 4^th ^postoperative day, the patient developed severe acute pancreatitis and was admitted to the Intensive Care Unit. She developed multiple organ failure and died on the 30^th ^postoperative day.

## Discussion

Few cases of adenocarcinoma of the third and/or fourth portion of the duodenum have been reported [3-11]. Causative factors have not been clearly identified [5]. Patients with familial adenomatous polyposis (FAP) and Gardner syndrome are considered to have a higher likelihood of developing duodenal cancer [12,13]. Patients who have duodenal polyps without a predisposing family history are also at an increased risk [5].

Adenocarcinoma of the third or fourth part of the duodenum presents a diagnostic challenge. Symptoms may be absent until the tumour has progressed leading to a delay, of several months, in presentation [5,7,8]. The most common presenting symptom is abdominal pain; other clinical manifestations are nausea, vomiting, weight loss, anemia, fatigue, weakness, gastrointestinal bleeding or obstruction [4,5,7-11]. Diagnosis is also often delayed due to the vague and non-specific symptoms and the subsequent difficulties in performing the relevant investigation, while most patients undergo a number of diagnostic tests before surgical exploration [2,5-8,11]. Moreover, Cunningham reported that preoperative diagnosis was obtained in 6 of 13 such tumours [8]. The majority of these tumours have infiltrated through the duodenal wall at presentation with many being irresectable due to local and distant invasion [1,5,7-11].

Diagnosis is usually made by upper gastrointestinal contrast study and endoscopy [4,5,7-11]. Their location, however, is often inaccessible to endoscopic viewing which may result in failure to diagnose them at endoscopy [5,8,10]. In some cases [6,8], US or CT findings have prompted repeat endoscopy with advancement deeper than usual, into the third and fourth duodenal portion, leading to diagnosis as in our patient. Patients may have at least one negative gastrointestinal contrast study before a positive result on a subsequent study [5]. Endoscopy with extra-long fibre optic scopes may be of benefit.

Abdominal US is helpful for diagnosis and evaluation of vascular involvement [6,8]. Lesions appear as irregularly marginated hypoechoic masses but tumours smaller than 2 cm may not be detected [6]. Contrast-enhanced CT scan is useful for diagnosis and determination of malignancy and resectability [4,5,8,10,14]; however, tumours smaller than 2 cm may not be seen [14]. Features indicating malignancy are an exophytic or intramural mass, central necrosis, and ulceration while entirely intraluminal location indicates a benign tumour [14]. These features, though, are sensitive but non-specific. Vascular encasement, invasion of contiguous organs other than the head of the pancreas, distant lymphadenopathy, or metastases precludes curative resection [14]. Endoscopic US and magnetic resonance imaging (MRI), although not frequently used so far, are useful for the diagnosis, staging, and determination of resectability of these tumours.

The treatment of choice is radical surgical resection [2,4-11]. The correct operation (pancreaticoduodenectomy, local excision or segmental resection) has been debated. Worldwide there is no general attitude on optimal surgical procedure in treatment of primary non-ampullary adenocarcinoma of the duodenum, especially for early stage disease. Due to its rarity, there is a lack of studies comparing local excision, segmental resection and Whipple procedure in the management of this neoplasm. Some authors prefer local excision or segmental resection while others duodenopancreatic resection, even in the case of early stage duodenal cancer with aim to avoid tumour recurrence, considering pancreaticoduodenectomy the procedure that satisfies the principles of an adequate curative cancer operation. Heniford [7], Santoro [9], and Barnes [10] reviewed 12, 33, and 67 patients with non-ampullary adenocarcinoma of the duodenum, respectively; no significant difference between pancreaticoduodenectomy, wide local excision or segmental resection was observed while tumour stage and resectability were the only predictive factors of survival identified. Moreover, in a study of 47 patients by Tocchi [5], no statistically significant difference was found between patients who underwent duodenal segmentectomy and those undergoing pancreaticoduodenectomy in terms of local recurrence, distant metastases, disease-free survival and overall survival. However, statistically significantly higher blood transfusion requirement, hospital stay, morbidity, and mortality were noted in the pancreaticoduodenectomy group. Factors influencing survival were TNM staging and, particularly, lymph node status. The authors suggested that duodenal segmentectomy may be preferred to pancreaticoduodenectomy because it is associated with low rates of morbidity and mortality, while exerting similar results [5]. It should be noted, though, that local recurrence after wide local excision of early stage disease has been reported [3]. Regardless of the type of surgery, curative resection results in a significant survival advantage compared to noncurative resection or nonoperative management [4,5,7-10]. In advanced disease, and particularly in duodenal obstruction, palliative resection, gastrojejunal bypass or duodenal stents may be indicated. In our case, segmental resection was performed. Our patient died due to severe acute pancreatitis leading to fatal systemic inflammatory response syndrome a complication that has also been reported by others [4].

Little is known about the use of radiotherapy and chemotherapy, but most physicians utilise therapeutic strategies modeled on the management of large bowel cancer [2]. Cunningham observed no significant benefit of adjuvant chemotherapy on survival [8]. The prognosis is generally poor and depends on stage at presentation and surgical resectability [2,5-10]. Prolonged survival following complete resection is possible while irresectable disease has a very poor prognosis [4,5,7-11]. Selective, individualised use of pancreaticoduodenectomy, wide local excision or segmental resection as surgical treatment options seems to provide a rational approach to this rare disease.

## Conclusion

Adenocarcinoma of the third and fourth part of the duodenum is very rare. Patients typically present with a long history of variable, vague symptoms and, many, with advanced disease. A higher degree of suspicion and a more aggressive, persistent investigation should lead to earlier treatment, higher curative resectability rate, and, therefore, better long-term results. The treatment of choice is radical surgical resection. The optimal surgical procedure, though, remains controversial. Multi-institutional prospective studies comparing pancreaticoduodenectomy with segmental resection or wide local excision as well as trials of chemotherapy and radiation therapy are needed to identify their impact on prognosis and suggest appropriate treatment recommendations.

## Competing interests

The authors declare that they have no competing interests.

## Authors' contributions

HM contributed to manuscript conception, research, acquisition of data, drafting and writing of the manuscript. DT contributed to organising and drafting of the manuscript, and critically revised the manuscript. KGT contributed to organising and drafting of the manuscript, and critically revised the manuscript. GG carried out the histopathologic evaluation and contributed to writing of the manuscript. SK contributed to organising, drafting and critical review of the manuscript. IB carried out the operation and contributed to acquisition of consent and critical review of the manuscript.

All authors read and approved the final manuscript.

## Consent

Written, informed consent was obtained from the daughter of the patient for publication of this case report and accompanying images. A copy of the written consent is available for review by the Editor-in-Chief of this journal.
